# Social networks, health and identity: exploring culturally embedded masculinity with the Pakistani community, West Midlands, UK

**DOI:** 10.1186/s12889-020-09504-9

**Published:** 2020-09-21

**Authors:** Farina Kokab, Sheila Greenfield, Antje Lindenmeyer, Manbinder Sidhu, Lynda Tait, Paramjit Gill

**Affiliations:** 1 Institute of Applied Health Research, The University of Birmingham, Edgbaston, Birmingham, B15 2TT UK; 2 School of Nursing, The University of Birmingham, Edgbaston, Birmingham, B15 2TT UK; 3 The Health Services Management Centre, University of Birmingham, Edgbaston, Birmingham, B15 2TT UK; 4 School of Health Sciences, The University of Nottingham, Nottingham, NG7 2RD UK; 5 Warwick Medical School, Warwick, Coventry, CV4 7AL UK

**Keywords:** Qualitative, Pakistani, Men, Social capital, Identity

## Abstract

**Background:**

Migrants from South Asia living in developed countries have an increased risk for developing cardiovascular disease (CVD), with limited research into underlying social causes.

**Methods:**

We used social capital as an interpretive lens to undertake analysis of exploratory qualitative interviews with three generations of at-risk migrant Pakistani men from the West Midlands, UK. Perceptions of social networks, trust, and cultural norms associated with access to healthcare (support and information) were the primary area of exploration.

**Results:**

Findings highlighted the role of social networks within religious or community spaces embedded as part of ethnic enclaves. Local Mosques and gyms remained key social spaces, where culturally specific gender differences played out within the context of a diaspora community, defined ways in which individuals navigated their social spheres and influenced members of their family and community on health and social behaviours.

**Conclusions:**

There are generational and age-based differences in how members use locations to access and develop social support for particular lifestyle choices. The pursuit of a healthier lifestyle varies across the diverse migrant community, determined by social hierarchies and socio-cultural factors. Living close to similar others can limit exposure to novel lifestyle choices and efforts need to be made to promote wider integration between communities and variety of locations catering to health and lifestyle.

## Background

The growing prevalence of obesity alongside co-morbidities such as diabetes and cardiovascular disease (CVD) require global preventative action [1]. At greater risk are migrants, where the movement of people across borders coincides with changing health beliefs and behaviours [2].

In the UK, the 10-year NHS plan highlighted the need for greater support in Black and Minority Ethnic (BAME) communities to address specific health needs through lifestyle services which subsequently led to increased funding to further develop the Diabetes Prevention Programme [3]. Given that 65% of men were classified as overweight or obese in 2016 (in the UK), there is a clear need to increase men’s access to lifestyle services [4].

Neighbourhood studies in the UK have shown minority-ethnic groups are over-represented in deprived areas characterised by disadvantaged physical environments, including inadequate leisure facilities, housing, and over stretched primary and secondary healthcare facilities [ 5, 6]. The worst neighbourhood environment for the White majority is comparatively better than that of minority-ethnic groups in the UK [6, 7].

### The Pakistani community

The South Asian diaspora is one of the largest migrant groups within high-income countries, with the largest community in Europe residing in the United Kingdom (1,174,983 out of 2,255,000 Pakistanis in Europe living in the UK) [8]. However, the health behaviours of this community reflect the patterns found in their ancestral countries of origin [9, 10]. Members of the migrant South Asian community in the UK, USA and Canada continue to have a high prevalence of diabetes and coronary heart disease (due to smoking, poor diet, alcohol and low physical activity levels), despite comparatively greater access to healthcare services in their host country [9, 11] . Currently, 7.4 million people in the UK are living with heart and circulatory disease, where one in seven men compared to one in 12 women die from coronary heart disease (CHD) [12]. However, over the past few decades there has been a decline in the mortality associated with CVD in the UK [12]. In addition, a study in Pakistan found that CHD, which is higher amongst men compared to women, contributes to the growing threat of CVD in South Asia [13]. Internationally, lower socio-economic status continues to be detrimental to non-White ethnic groups with regard to their susceptibility to CHD compared to their White counterparts [14]. For example, Pakistanis living in the UK continue to live in areas of greater deprivation than their Indian counterparts [14] and have great risk for developing CHD (8 and 6% respectively) [15].

In part, this is a reflection of how healthcare campaigns and local policies have failed to incorporate measures to support interaction and integration of migrant, minority-ethnic community members within preventative healthcare services [16].

Furthermore, health and social policies fail to consider the nuanced socio-cultural and religious differences that influence engagement with healthcare providers and access to healthcare information [ 17]. As a heterogeneous group, there are marked differences in the prevalence of illness and disease within the South Asian diaspora [18]. Compared to Indian men, the risk of CVD, coronary heart disease (CHD), diabetes and associated mortality is higher amongst Pakistani men and they are more likely to live in some of the most socio-economically deprived areas of the UK, with low income, poor educational attainment, and continue to face discrimination [15, 19].

Specifically, mass migration to the UK, from the province of Kashmir in Pakistan during the 1960s, has resulted in a community comprised of first-generation migrants as well as a growing young cohort of second and third-generation descendants who are trying to accommodate non-traditional, Western practices while balancing acculturation and their migrant status [20]. Often, they live in inner-city and urban former industrial areas such as the West Midlands in concentrated settlements i.e. ethnic enclaves [21–23].

### Social capital and migrant health

Research has highlighted the importance of social networks and network-based resources in the management of health conditions and development of self-care support [24]. In terms of migration, recent migrants can benefit from the security that social networks provide and build on existing social capital within microcosms, that can help find work, housing and support that is not available elsewhere [25]. However, there has been debate around the positive ‘buffering’ effect on health due to living in residential areas with a high ethnic minority concentration [26], that provides protection from racism through increased social Involvement in social networks may be negatively influenced by low socio-economic status that undermines community networks and relationships [27].

In order to better understand the socio-cultural factors relevant to these groups and how they influence health behaviours, the theory of social capital was applied. The theory of social capital can facilitate an understanding of how social networks and resources within an individual’s community (physical and social resources) can contribute towards shaping health beliefs and behaviours. There are several definitions of social capital, but for the purpose of our research, we understand social capital as “resources embedded in and acquired from social networks and interactions based on connecting ties, trust and reciprocity, through which members of a collective can attain various ends or outcomes that are of benefit for the individual/collective” [25, 27]. At an individual level, social capital provides a pool of resources from other members of the social network that the individual belongs to [28]. For healthcare, the social network is a tool to access the social capital to obtain information about health behaviours, support to undertake lifestyle change (diet or exercise) and justify said behaviour [29]. Social networks are structures that can provide individuals with ‘companionship, advice, emotional support and practical assistance’ [30].

A review of social capital literature identified a lack of consistency in the operationalisation of social capital where migrants, even those having high socio-economic backgrounds, can lack bridging social capital due to limited connections with local people [31]. Within social networks or ties, individuals can form bonds (with similar) and bridges (with different) members of the neighbourhood. Networking within the local neighbourhood can be beneficial; research on social structures within communities in the Netherlands noted positive associations between neighbourhood-based social capital and life satisfaction for residents who spend significant time in their community [32]. A mixed-method study, guided by Bourdieu’s theory of practice, found that the resources necessary to create social capital (cultural capital, ability to socially network) differ according to the socio-economic status of the neighbourhood, but living in affluent areas did not guarantee access to beneficial social networks [33]. However, there is limited research on networking within minority-ethnic groups who are often mobile and have a more dynamic perception of community, neighbourhood and social capital [34].

There is an assumption that individuals from minority ethnic groups have the appropriate social or familial support to manage or prevent health conditions [35], where a lack of social capital could influence the health inequalities faced by members of the Pakistani community. Links between social capital and self-care are seldom researched in relation to chronic disease management but could indicate the importance of developing network-centred approaches for engaging socially and economically deprived groups [36]. Research exploring cardiac rehabilitation, physical activity and CHD in the South Asian community have uncovered gender based socio-cultural barriers for women, and the role of empowerment, communication, relationships and the environment [37–40].

Pakistani Muslim men’s ‘relational, emotional and intimate dimensions’ are underexplored. However, research by Britton (2019) with Muslim families highlighted the shifting gender and generational relations, in particular, changing masculine roles as a consequence of racism and marginalisation due to socio-political concerns such as ‘islamophobia’ and stereotyping of Pakistani Muslim men after incidents such as the ‘Rotherham grooming’ cases [41]. The ‘Rotherham child exploitation scandal’ consisted of organised child sexual abuse in the northern English town of Rotherham predominantly by British-Pakistani men and the media framing had an adverse impact on the Muslim community [42]. Pakistani men face social constraints and isolation (similar to other Muslim ethnic minority men who are not Pakistani) within work environments due to cultural differences resulting in limited networking opportunities. At times, Pakistani men reject mainstream identities especially when living in socio-economically deprived areas (‘ghettos’) [17, 43].

Within the context of our research, social capital, in particular, social networks, are viewed as the mechanisms which Pakistani men can use to achieve their desired health goals. For these men, there are fundamental factors including culturally embedded patriarchy and the generally strong ethos for practising Islam (that can vary between families and individuals) intertwined with the need to maintain certain cultural norms when living in areas of high minority-ethnic density that influence the chances of achieving health goals [44, 45].

However, there is insufficient research explicitly exploring social support concerning health beliefs and lifestyle choices using an interpretive theoretical lens, especially where the focus is on one minority ethnic community, such as the Pakistani community, rather than a mixed-ethnic cohort (South Asian).

## Method

### Aim

We explore migrant Pakistani men’s views on health behaviours related to CVD prevention (diet and exercise) and social support for achieving these behaviours within the local community. Social capital is used as an interpretive lens to understand how social networks, trust and cultural norms can shape Pakistani men’s access to health information and support a healthy lifestyle.

### Design

A community-based interpretive qualitative study with in-depth, face-to-face interviews, and completion of the convoy model diagram to help illustrate individual social networks [46].

### Setting

Participants were recruited during October 2013–February 2014 from across the West Midlands and in particular Birmingham. Birmingham has the highest concentration of South Asian individuals (13.4% of the total population of South Asians living in the UK) compared to other parts of the UK, with the largest non-White group being Pakistani (15.4% of minority ethnic groups) [47, 48].

### Sampling, access and recruitment

Issues such as literacy, language, translation, knowledge of research, cultural advocacy for taking part in the research (trust and a sense of belonging), obtaining consent, as well as transportation to research facilities were taken into consideration [49]. In the context of our research, ethnic concordance with the researcher encouraged involvement and shared understanding through a shared language and socio-cultural background [50–53].

Participants were recruited using purposive sampling through community businesses that were spatially proximal to members of the Pakistani community [54]. The business areas acted as enabling places and encouraged participation [55]. We approached a selection of business districts in Birmingham due to their strong ties with members of the local Pakistani community, who acted as ‘gate-keepers’ and ‘Research advocates’. For this project, ‘Research advocates’ were defined as members of the community who were willing to promote recruitment and participation through their social connections and build rapport with members of the local community. Word-of-mouth advertising, third sector organisations, social media (i.e. Facebook) and snowballing [56] were used in conjunction with the businesses. The study information was presented to participants using 1) information sheets, 2) oral advertisements, 3) lay-led posters and 4) informative ‘posts’ sent through local volunteering services emailing lists.

### Participants

Participants’ characteristics are outlined in Table 1.

**Table 1:** Characteristics of male participants

Generation	Age 18–29	Age 30–49	Age 50 and over	IMD
First (*n* = 8)	2	2	4	5: *n* = 6 4: *n* = 1 3: n = 1
Second (*n* = 12)	8	3	0	5: *n* = 10 4: *n* = 2
Third (n = 1)	1			5: n = 1

Index of Multiple Deprivation (IMD) level of 1 = least and 5 = most deprivation

First generation (Born in the subcontinent migrated directly/indirectly to the UK)

Second generation (Born in the UK or received formal education from the age of 5)

Third generation (Born in the UK and at least one parent from the second-generation)

### Developing the topic guide

Interviews were designed to explore the influence of social networks, trust and cultural norms (as social capital) in forming health behaviours related to preventing cardiovascular disease [57]. The topic guide was piloted with four voluntary participants from the community, who were approached to take part in interviews prior to recruitment for the main study, to explore its strengths and weaknesses and the ability to translate questions into Urdu (the language predominantly spoken in Pakistan) [58]. As a response to feedback from participants, the topic guide was further developed to avoid dichotomous responses in Urdu as some questions became close-ended when translated. Consequently, the piloted topic guide included components of the social capital theory including social networks, trust and cultural norms with changes that better suited the nature of this investigation as it ‘outlined key issues and subtopics to be explored with the participants’ [59]. A copy of the interview guide can be provided upon request.

### Data collection

Participants were invited to take part in a one-to-one interview that would last an hour in a location of their choice (home, work, business place they were recruited from, or at the University) and in their preferred language (English, Urdu or Punjabi). The interview process involved introducing the study (verbally and/or giving an information sheet, in Urdu or English), completing the Convoy model diagram [60], and the interview. The Convoy model proposed by Kahn and Antonucci (1980) is a framework for observing social network development over time [60]. The diagram consists of a series of concentric circles, with the individual at the centre and the importance and proximity decreasing with each progressing circle. Participants were asked to write their name in the centre of their circles and then place the names of individuals (e.g. mother or sister) based on the importance of that relationship. The names of relationships were placed on one of three circles, where the first innermost circle was for the most important relationships, and with each progressing circle the relationships became less important. Although, the Convoy model was designed to collect data on the effects of ageing on social networks, it has been applied to other contexts such as accessing health information and support [61]. The diagram has also been used to explore components of social capital to understand better the relationship between social networks, across age (younger and older), socioeconomic status and lifestyle choices [40, 62]. Our purpose was to elicit a personalised illustration of relationship networks, achieve conceptual equivalence (same understanding of a concept in all languages) of different terms (e.g. social networks) and use it to elicit discussions on social relationships and trust. The model was used purely to elicit discussion on social networks, irrespective of participant age. At times participants referred to websites and images on their phones to illustrate their points (without prompt).

### Ethics

All data were stored in accordance with University of Birmingham ethical guidelines. Ethical approval was given by The University of Birmingham Science, Technology, Engineering, and Mathematics Ethical Review Committee application number: ERN_13–0450. All participants provided written consent to take part in the research and were informed of their right to withdraw.

### Analysis

Data collection was an on-going and iterative process where transcribing and coding occurred simultaneously until saturation was reached [63].

The interviews were carried out between October 2013 and February 2014, audio-taped on encrypted recorders, transcribed verbatim (as well as anonymised), and where necessary translated into English (by FK and checked by MS and PG) [64, 65]. NVivo 11 software was used to manage the data during analysis. A South Asian researcher based at the University of Birmingham verified samples of the transcripts for conceptual equivalence, and to ensure accuracy and quality of translated material.

**Fig. 1:**
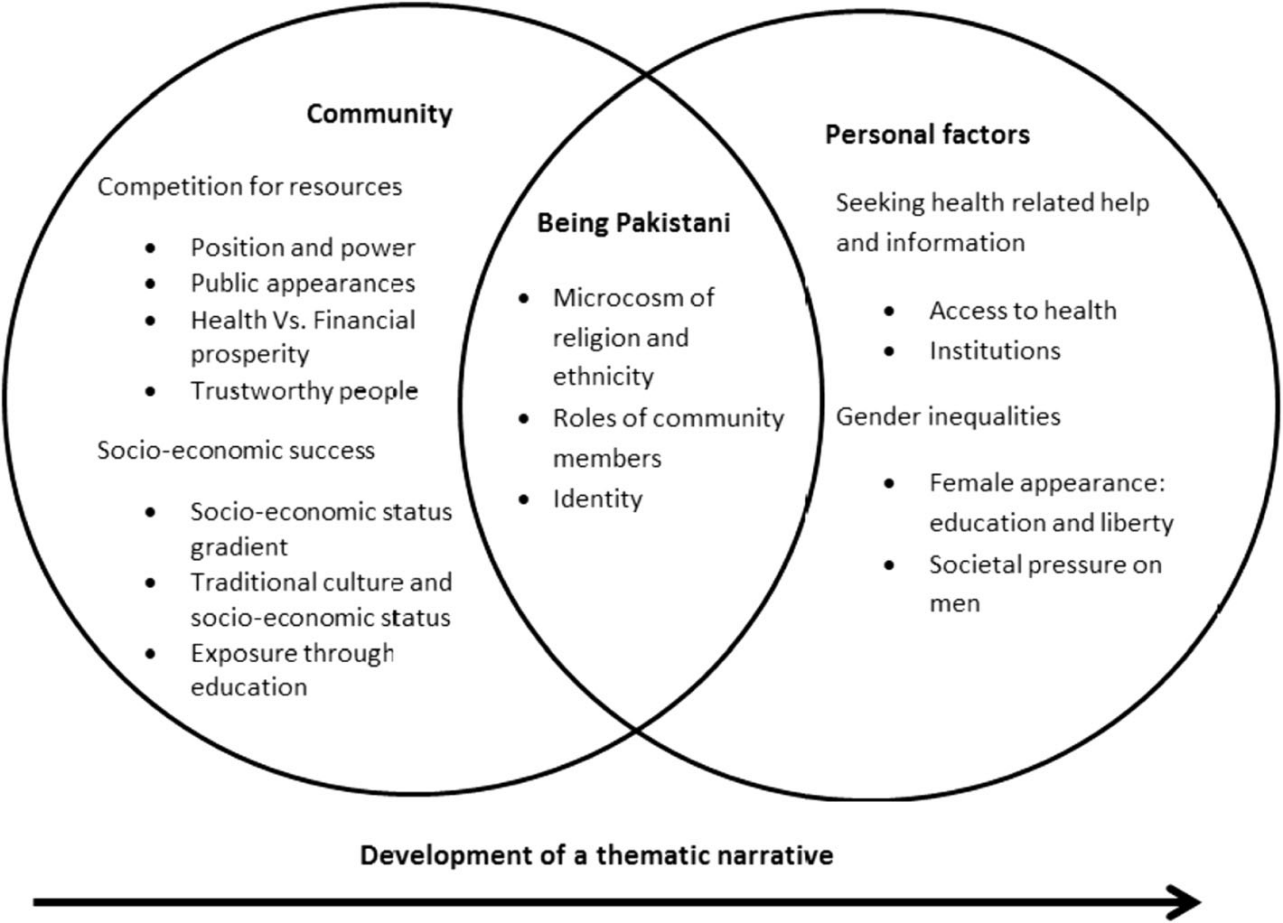
Development of the thematic narrative (communal to personal perspective)

The framework method was used to provide a transparent outline of the analysis based on typologies (age, gender and generation), concepts, and differences and similarities [66]. The researchers worked through five stages of analysis: 1) familiarisation with the data, 2) identification of a thematic framework, 3) indexing (systematic application of the coding framework), 4) charting (creating charts with participant demographics and data), and 5) mapping and interpretation [67]. Data reduction was achieved by systematically placing the data into manageable charts as a process of data management and reduction [68]. The major themes were used to categorise the frameworks as functional categories. Figure 1 illustrates the development of themes into categories.

## Results

The views of 21 tri-generational men from different educational and occupational backgrounds were analysed to understand men’s perceptions of social networks and access to health support from the Pakistani migrant community across the West Midlands, UK. An outline of the different areas of the West Midlands participants were recruited from is given in Table 2.

**Table 2:** Outline of the different areas in the West Midlands

Location in Birmingham (constituency, ward and area)	Description	Population total	Pakistani population
Area A	Centre of industrial revolution in the Black Country	312,952	10,399
Area B, G, J and K	Multi-racial residential area with a diverse range of shops, contemporary restaurants and cafes	115,904	37,653
Area C	Middle-class residential area, Southwest of the city-centre, leafy parks and cricket ground	96,568	2844
Area D, I and L	One of the main centres for the Pakistani community but trends for moving outwards	126,693	19,484
Area E, F and H	Predominantly Pakistani (Mirpuri inhabitants) characterised by take-away restaurants and ethnic-specific shops	121,678	46,042
Area M	Majority non-White, close to the city-centre, occupied largely by West Indian and South Asian Immigrants	107,090	12,902

[69–72]

Findings of this research will be presented by illustrating participants’ views of their local area as a Pakistani microcosm and ethnic enclave, with two prominent social spaces of influence: The Mosque and the gym. Perceptions and pluralism centred around identity but were also generationally interpreted. Although all participants identified as Pakistani, self-perception was also nuanced along the lines of contrasts between Pakistani, Eastern norms with British and Western culture throughout the themes, where at times participants identified themselves as British Pakistanis and the internal conflict this brings.

### Pakistani microcosm

Pakistani microcosms in Britain can be distinct in terms of their ‘rules, standards, faith, community, economy, and reality’ [73]. Within our research, participants noted how their local environment played an essential role in reinforcing traditional Pakistani norms and forming identities. The first-generation participants’ narratives centred around identity and perceptions, which shed light on generational divisions.

For example;

“I used to think that once the first-generation immigrants have done their time, the next generation will be better, but unfortunately I see they are actually worse, because, their living style is different, they look distinctly different, if you look at them and their style of, the beard and all that there’s single line running from there and there (gesturing to face), you don’t see that in anybody else so its specific to Asian young men, the look they have. They speak different, it’s not a, a proper language...its slang lingo which they use”

(50+ years, first-generation, IMD 3, area B)

“The most important thing in Pakistani culture is that women have to be covered. So, most sports women are not fully covered, so that is a big decision for the family to take. So as long as the woman who is playing, if she is playing some sport and she is fully covered then I think she should be allowed to play”

(18–29 years, first-generation, IMD 5, area E)

One of the participants further provided insight into how it is possible to better navigate any tensions by accommodating Western and Eastern norms alongside each other while reflecting positively on successful acculturation into the UK.

“So, integration has never been an issue: we have maintained the good qualities of our cultural background. We have adopted lots of very, very good things, which is in this culture (British). So, I would say that is a hybrid”

(50+ years, first-generation, IMD 3, area B)

Political and socio-spatial aspects of living in a majority migrant environment affected the development of individual identities, where living in an area with high ethnic-density (i.e. that participants described as a social world defined by ‘Pakistani’ values) increased exposure to influences strengthening the civic notions of being Pakistani. Despite any perceived sense of security associated with living in a predominantly Pakistani area, some second-generation men felt their parents’ decision to live in such areas limited exposure to other communities, cultures and social practices.

“The thing they (parents) lacked were mixing, living in the English lifestyle ‘cos they weren’t taught to do that and bringing up their children, English children. You can’t be a Pakistani from Pakistan; your children have to be brought up like the British”

(30–49 years, second-generation, IMD 4, area H)

Limited integration was viewed as a disadvantage by some participants, concerning their social development, as they vocalised the effects of limited integration and understanding of British lifestyle choices due to participants’ concerns over losing their migrant cultural-religious identity [74].

#### Social mobility and traditional values

Whereas first-generation men were preoccupied with labour intensive jobs and settlement, second-generation community members justified their need to be competitive and fulfil their aspirations to develop beyond their ‘migrant’ status and show trans-generational growth, e.g. by challenging stereotypical views of unhealthy and overweight Pakistani men.

“They’ve had their kids, they’ve bought their properties, they’re brought their property in Pakistan; they’ve done enough now. I don’t think they strive to live to 90 years or 100 years. I think they live to 70 and 80 years and then they’re happy and that’s the difference”

(30-49 years, second-generation, IMD 4, area H)

Community members were conscious of deviating from the traditional extended family model and associated norms by aiming to increase monetary capital and accessing diverse social networks. This could cause tensions between first- and second-generation men as lifestyle choices seen as ‘modern’ behaviours were associated with socio-economic success and a disregard for family values.

“You’ll have the secular types, the families who are very professional, who are, you know, earning 40-50k a year, his wife will be professional as well, I don’t think they will be, [don’t] do too much looking after their elders, personally, ones with traditional values, yes! But certainly, modern values, no”

(30–49 years, second-generation, IMD 5, area B)

Upward social mobility towards new spaces and away from community norms in ethnic enclaves was viewed as an opportunity to limit exposure to unhealthy lifestyle choices. Living or being close to takeaways could lead to excessive calorie intake and limited physical activity in lower-income and deprived communities [75].

“Go to (location Site A) and that’s seen as upper class. So, you will not find a takeaway for a good stretch of a mile or two. Naturally, more likely than not, because of their daily routine, most of the people working there are working 9-5, 9-6. So, most of their food is self-prepared, right, whereas our food, well, if we don’t like it, you’ve got a chip shop right just there, ready-made for you … just what’s present in the community is having an influence on your health” (18–29 years, second-generation, IMD 5, area E)

The limited socio-economic development of some members of the Pakistani community was further demonstrated in the ability of younger generations to afford to adapt their diet to suit their physical lifestyles. The narratives of moving away are intertwined with creating distance from cultural pressures and opening up to more diverse networks. At times, second-generation men chose non-traditional meals and routines to achieve their personal goals of looking masculine. The aforementioned quote also illuminates the disparity between participants from lower and more middle socio-economic status backgrounds. Participants who were adopting more middle-class values were moving away from local spaces and traditional migrant beliefs, whereas younger, working class participants were taking more ownership over spaces and trying to change the meanings, values, and beliefs in deprived communities whilst trying to efficiently utilise their existing social networks to improve their lifestyles. As some participants were recruited from areas with high levels of deprivation, it was apparent that income and occupation was associated with access to food options and related to lifestyle patterns.

#### Health and social needs of first-generation men

Health spaces where members of the community can obtain healthcare and lifestyle knowledge or information (e.g. on diet and exercise) are age and generation-centric based on the different needs of older, younger and recently arrived migrant men. When outlining the key institutions that played a role in their physical, emotional and social well-being, Pakistani men noted the General Practitioner’s (GP) surgery as an important source of information for first-generation men.

Generational differences were also reflected in the health-seeking behaviour of first-generation and older migrant participants who relied on the information provided by their GPs; especially when seeking assurance.

“The doctor that I know he treats me very well, he gives me medicines, anything that I need to get I get in a nice way”

(50+ years, first-generation, IMD 5, area B)

Second-generation men did not share these views and were more dissatisfied by the approach used by their GP, resulting in them relying on alternative sources of information; predominately, online resources and through social networks.

“I think going to the gym has been a big contributing factor with that because you meet other individuals who you see, they’ve achieved good physical shape and you ask them for tips. You go online you do research, and obviously they just do that at work, in a lot of things that I’ve learned through academia made me aware of what things I need to look out for, what diseases there are out there and how they develop. So, I think, obviously self-education is key but all that comes through participating in these kind of activities, i.e. going to the gym, playing sports and, that’s how you come across things”

(18–29 years, second-generation, IMD 5, area I)

For some first-generation and older men, it was recognised that the Mosque, rather than the local gym, promoted integration within the community.

“Baby steps, Rome wasn’t built in a day … it’s about investing that time in individuals not as cattle or herd, do you know who’s got a big role to play in all this? Our own community, you and I know that the public services and strategy organisations aren’t funding, and sources are very limited, our Mosques and our Gurdwaras our Hindu temples have got a big role to play in this”

(30–49 year, second-generation, IMD 5, area B)

Although, at times, Pakistani men of different ages and generations may be using the same health space, what is considered a health space or not differs. For example, for older men, the Mosque was considered a location for promoting dialogue and raising awareness surrounding community well-being and health.

### Ethnic enclaves and Pakistani patriarchy

First- and second-generation participants commented on the high level of perceived security threats within (inter-familial conflicts) and outside (discrimination) of their community as a motivation for monitoring and controlling women’s behaviour. By sensationalising stories of women being harmed in the wider community, men could emphasise the need for their authority and vigilance.

“My sister was racially attacked, years ago. My elder sister, she used to cover (hijab) and some Black guys attacked her. She phoned me; I didn’t get there in time, but my friends did though”

(30–49 years, second-generation, IMD 4, area H)

Masculinity within the context of British Pakistanis’ lives was reinforced in men’s attitudes towards women. Men felt a degree of responsibility in determining the level of safety in the local area for women to socialise or exercise in. Safety meant protection from physical but also ideological threats through limiting exposure to unfamiliar or novel lifestyle practices. These views also included gender perceptions, where women are expected to remain slim in maidenhood to successfully obtain a suitable spouse.

“There’s more pressure on girls and I think that’s what causes them to be more health conscious. I’m not saying its good pressure, but that is one of the ways, ‘cos unfortunately, society, you know … a girl gains a bit of weight she’s seen as unattractive or whatever, whereas a guy, even with a bit of weight if he’s doing alright (financially) in life, he’s very much attractive” (18–29 years, second-generation, IMD 5, area I)

Men recognised the barriers that were placed on women and the limited opportunities that were offered to them. Younger men may not actively enforce patriarchal values but recognise the role they play to inhibit women’s health and well-being while supporting men within the community.

“… it’s hard for the community to see women go to the gym, I think they’re just expected to not really concentrate on their health much. In some cases, guys get food cooked for them from the family (if) they want to go to the gym and work out, but if that was a girl, I don’t think that would be the same case”

(18–29 years, second-generation, IMD 5, area E)

Living in a particular area further perpetuated certain expectations and gender-based roles that were difficult to negate.

### Young men’s territory

#### Second-generation men and the gym

Younger men preferred the gym as a social space, which they viewed as a mechanism for developing their status within the community (as providers, leaders, and protectors), access to social networks and develop physical strength, where health was not necessarily the main concern but a secondary outcome.

“I don’t think they do it for health reasons because they’re- people wanna go to the gym and they can do it just- they want to do it, to look good in a t-shirt”

(18–29 years, second-generation, IMD 4, area H)

This participant notes that the gym provided individuals with the opportunity to shape their physique, to look a particular way for social gratification. Consequently, some men were territorial about their workout space, which they deemed unsuitable for older men and in particular women.

“If she takes that first step, she goes in, and there’s a lot of sweaty guys training, making all sorts of noises, it’s uncomfortable. Whereas, if you went to (brand gym), it’s normal to have people of all ranges and ages and colour … whereas you come in to my gym and you see fat, hairy guys, and you’re sweating ‘argh’ (grunting noises), the girls are not gonna want to be there …”

(18–29 years, male, second-generation, IMD 5, area E)

This participant’s views on the gym encapsulate community sentiments on gender and generational differences on how individuals socialise within socially segregated spaces. The gym is a space designated for young men to shape their bodies but also develop their thinking towards social matters and issues. The nature and level of support available within the health space were targeted at young Pakistani men to develop their social capital by forming connections with others from a similar age and generation; who shared their experiences of growing up in a British Pakistani community and its encompassing cultural norms.

Again, territorial views on ‘Asian gyms’ as spaces that facilitated Pakistani men’s social and physical goals contribute to the dialogue on socially segregated spaces and how they can shape contemporary cultural norms:

“If you go to a White persons’ gym, everything is in the same room, if you go to an Asian man’s gym you’ve got weights and weights and weights, and you’ll have a little room in the corner … it’ll have like three treadmills”

(18–29 years, second-generation, IMD 5, area E)

Working out in local gyms in comparison to branded facilities also aligned with the formation of identity as one of the local ‘meatheads’, perceptions of socio-economic status (men working hard to appear physically strong despite their socio-economic status) and perceived level of acceptance. This reflects more traditional Pakistani or Indian Punjabi perceptions of masculinity based on physical strength [52]. Some participants felt more comfortable focusing on developing a muscular physique in the local gym rather than the more holistic approaches adapted by bigger brands.

“‘Meatheads’ meaning the big bulky guys who actually, more of a guy’s gym rather than the normal health club that you’d expect, like women or stuff like that. They wouldn’t be there. It’s normally a guy’s gym”

(18–29 years, third-generation, IMD 5, area E)

Consequently, some participants who could afford to avoid these health-related spaces described their discomfort with them;

“I just didn’t feel good in that environment. Like, there was a lot of people, the way they just talked to each other, there was a lot of bad words being said, and you could sometimes smell them doing drugs or, it just wasn’t a nice environment”

(18–29 years, second-generation, IMD 5, area E)

#### Physical and moral strength

The gym was an arena where men shared their knowledge and experiences on how to stay fit and continue being exemplary, young Pakistani men. It became apparent that the use of their local gym as a source of inspiration, socialisation, and contest for a masculine physique contributed to the desire to be an affluent member of the community as reflected in the rivalry participants expressed in terms of their physical appearance (demonstrating masculine strength and morals). Men with a larger and stronger, socially desirable alpha-male physique were seen as role models and better suited to hold positions of authority or leadership in their family or community.

“I’ve got the broad shoulders to make decisions, whether the family likes them or not, and that’s the role I play in my family”

(30–49 years, second-generation, IMD 4, area H)

Men developing their physical strength was a demonstration of their commitment to protecting their family and their community’s cultural values and norms. In some instances, protecting (defending) the cultural norms of the community was taken literally.

“The weaker you are the more vulnerable you are to other people, the more prone you are to being attacked, so the more healthy you are you can protect yourself, you can protect your family, if you get into a fight or something and you are weak, its most likely that the person that’s fighting you will be stronger than you … protection, protecting yourself, protecting your women, protecting your children, I think that is a big motivation (for going to the gym or being healthy)”

(18-29 years, first-generation, IMD 5, area E)

Despite their perceived status as protectors, Pakistani men could not infringe upon the ‘tight’ (established) cultural norms in the community that dictated social or personal behaviour. Consequently, ‘deviant’ or *haram* behaviour could negatively affect an individual’s position within their community networks and competition to remaining in a superior authoritative (masculine) role. The desire to be an influential (physically and morally) figurehead required a balance between adopting Western behaviours (going to the gym and alternative diet) and a vigilant moral compass (against behaviours that challenge the community’s norms). Pakistani men were self-conscious of how quickly they could come under scrutiny from one another in this rivalry for leadership.

“The younger generation at the moment is hot headed, and once, if they see you in good shape or that you have a good body, they get jealous, like, ‘look at him, he has a good body, we don’t’ and even an argument can start over a petty thing”

(18–29 years, first-generation, IMD 5, area E)

Interviewees felt that young men competed with each other for a stronger physique, and not just to exert their power and dominance (control) over their family members (related to being their protector).

## Discussion

Our study shows how second-generation Pakistanis in pursuit of professional careers or higher education were more likely to be exposed to diverse groups of people, and consequently have greater exposure to alternative lifestyle choices and opportunities to seek out healthcare information and support. However, second-generation descendants of migrants continued to feel disadvantaged in their ability to access potential networks of information and support due to the disparity between their experiences and that of the wider population, especially if their parents are migrants and/or speak a foreign language at home [76]. These patterns of behaviour amongst second or third-generation community members are not unique to the Pakistani community, as generational shifts are common amongst migrants when negotiating cultural or religious ambiguity amongst other ethnic communities. Generational differences do not only have the potential to influence shifts in cultural norms but can also affect engagement and citizenship where alternative social and political opportunities are available [77]. For example, second-generation Swedish-Sikh immigrants have struggled with different arenas of power (religious, traditional and cultural) to adopt religious norms and individual interpretations into networking opportunities within their wider social networks, e.g. self-help activities for the youth at the Gurdwara that contain contemporary issues within a traditional setting [78].

Our findings contribute to the current understanding of the role of social motivators (such as competition for an alpha-male position) in the Pakistani community e.g. when going to the gym and moving beyond the pursuit of better physical health towards appearance in the community [79]. Most notably, Pakistani men viewed their local gyms (that were run by other Pakistani men in the community) as the ideal location to socialise within and familiarise themselves with the community’s norms. The role the gym played as a social space for men to define their masculinity can be linked to how perceptions of masculinity are associated with traditional masculine behaviours, such as health benefits and a stronger gender identity to accumulate ‘masculine capital’ [80]. ‘Masculine capital’ incorporates the skill sets and cultural competence required for men to fulfil social expectations in society [81]. Second-generation men dominated their social networks through their display of physical strength; stronger men were more likely to be approached for advice and support because their size was an indicator of i) access to wider community resources e.g. the gym, ii) being able to afford an expensive, high protein diet, and iii) having knowledge of health benefits i.e. nutrition and exercise techniques.

The gym was clearly marked as a male health space. Men used social spaces to define socio-culturally and religiously appropriate behaviours for women in their families. The impact of strong ‘hegemonic masculinity’ within the home during a decline in professional status at work has been noted [81], where the role of race, ethnicity and socio-cultural influences affect the formation of masculine identities [82, 83]. Making an association between ‘Asian’/Pakistani/‘meathead’ gyms to masculinity and being working class was contrasted with the ‘brand’ gyms for White men, women and a focus on health rather than appearance as a way of reinforcing their masculinity and control over their environment. Men from minority communities acquire dominance through responsibility and physical strength, which may lead to subordinating women in the community [84]. Expectations for women were not limited to the use of social spaces but included women’s physical and moral appearance. Women were expected to exercise in their homes, dress modestly when in community spaces (to preserve cultural values), compared to men who can socialise outside of the familial vacuum. This behaviour created a hierarchy in the community, where greater agency is given to men for how women should behave and how to influence the local community spaces to reflect this.

Being in close proximity to similar others, participants felt they were constantly critiquing each other concerning culturally acceptable behaviours. As members of the Pakistani community tend to live in areas with a high concentration of ethnic minorities and polarised enclaves, there is a higher tendency for conflicts to arise [85–87]. This form of ‘ghettoization’/segregation can adversely affect the lived experience within communities, with limited social capital and exposure to wider community settings [88].

### Limitations

Our findings present a ‘snapshot’, of Pakistani men living in the West Midlands, UK. How local health facilities are accessed or cater to for male health trends in the Pakistani community, should not be generalised to a wider population. However, findings may be transferable to other BAME groups. The present research provides insight into a migrant community in Britain, which may be transferable to other migrant populations in high-income-countries. A further limitation of the study was the inability to incorporate the multi-media data shared by participants (provided with no prompt) in order to protect their identity. Participants illustrated their experiences and described their social networks using photographs and websites in a bid to help the interviewer understand their lifestyle choices. Further research incorporating images to elicit responses and develop dialogue, using ‘Photovoice’ or ‘photo-elicitation’ methods, may be beneficial [89, 90]. Additionally, a greater effort should be made to include third-generation participants due to their limited representation in research generally.

A reflexive approach was taken to consider the influence of a female interviewer completing interviews with Pakistani men. The multi-disciplinary research team (consisting of men and women from psychology, medical sociology, and clinical backgrounds, and of South Asian, British and European descent) contributed to the analysis and interpretation of the findings to minimise bias. Data collection was undertaken until saturation [91].

### Implications for health (promotion/practice)

Recommendations can be made for better integration between primary and community care, and the uptake of health services around lifestyle changes in non-traditional health settings. Healthcare providers can adopt greater sensitivity towards socio-cultural barriers that are faced by migrant community members while trying to incorporate an understanding of personal identity and lifestyle choices [53]. Acculturation amongst second or third-generation migrants may vary and affect their use of community and online health resources, whereby recommended (e.g. NHS) websites can be promoted [92]. Furthermore, an overall holistic approach to healthcare that involves family members and locally recruited staff may develop social capital amongst community members, reduce isolation and increase confidence to make healthier lifestyle choices [93, 94]. The Mosque and gym are community spaces that can be involved in health outreach activities and should be considered when designing interventions or public health campaigns that seek to engage with different members of the community; especially first-generation or younger Pakistani men.

## Conclusion

Faith centred locations, such as Mosques, are prestigious social institutions that provide an opportunity to address some of the issues surrounding support and knowledge; however, not all community members (such as younger, second-generation men or women) feel this is currently appropriate. An effort to develop centres or advocates to promote alternative, culturally appropriate lifestyle choices could support the pursuit of a healthier lifestyle across diverse migrant community populations while encouraging social engagement. Nevertheless, there is a hierarchy of socio-cultural factors that influence the formation of lifestyle choices, where health may not be a priority and trust is required to build novel support networks.

## Supplementary information

**Additional file 1.** Interview guide. A copy of the interview guide can be provided upon request.

## Data Availability

The datasets generated and analysed during the current study are not publicly available due to concerns over anonymity and confidentiality, but some information is available from the corresponding author on reasonable request.

## Abbreviations

BAME: Black and minority ethnic
CVD: Cardiovascular disease
CHD: Coronary heart disease
GP: General practitioner
NHS: National Health Service
UK: United Kingdom
USA: United States of America
