# Supplementary material for: Social networks, health and identity: exploring culturally embedded masculinity with the Pakistani community, West Midlands, UK
Source: BMC Public Health. 2020 Sep 21;20:1432. doi: 10.1186/s12889-020-09504-9 (PMC7507824; doi:10.1186/s12889-020-09504-9)
Supplement: Supplementary file 1 — Additional file 1. Interview guide. A copy of the interview guide can be provided upon request. [file 12889_2020_9504_MOESM1_ESM.docx]

**Interview guide**

(Information in brackets are my thoughts/points to raise during the interview to engage with participants)

- Introduction: What we will be discussing (relationships, health goals, engagement with community and health services)
- Definitions**:** Social networks, health goals
- Demographic: Age, occupation, time spent in England, who do you live with, postcode

General health

1. How would you describe your health at the moment?

(How participants view their health and if they are satisfied)

Sub-questions:

- Please describe/outlines your daily routine/normal health practices?
- What is your current/future/ ideal health like?
- How do you engage with services/community in relation to health?
- What are your desired improvements for your health?
- Are there any social (family/community) facilities that are affecting your health? (If yes – in what way?)

Diet and Exercise

1. How do you monitor your diet and exercise?

(Personalised approach or based on lifestyle or routine of others)

Sub-questions:

- In your opinion what is a healthy lifestyle? Which people play a role in it?
- Do your friends and family agree or have different views?
- What kind of support do you receive/need to make health choices?
- What do you think affects your current/ future/ ideal diet or exercise plan?
- Any health targets/goals/ways in which you would change your diet?

Social relationships (trust, network ties, and cultural norms)

(What your relationship is like with people within/outside of your social circle and how they impact your lifestyle choices i.e. places you go to eat, activities you do together)

1. Who do you relate to when you think about your health?

Sub-questions:

- Who looks after you/ who do you look after?
- Which people do you approach about health? Why these people?
- How do people treat food or their diet?
- How does your social circle provide support or information you need when making lifestyle choices?
- How would these people react if you were to change your current lifestyle?
- Which activities would they like/not like you to do? Why?
- How do you accommodate certain social expectations when creating a daily routine? Which activities do you avoid?
- How likely is the opinion of others to influence your food or exercise choices?

Social capital (particularly civic engagement)

1. How do you engage with members of the community? Where? How often?

(How trust is built with others, do people share their lifestyle routines openly? Do they actively look up any diet/exercise programmes?)

Sub-questions:

- Are there any social activities that you think encourage a healthy lifestyle?
- How comfortable would you be in carrying out exercise or changing your diet around people in your community?
- Can you describe any community activities related to diet and exercise?
- From the people in your social circles who would you approach to discuss health goals?
- Are there certain people you rely on more than others?
- How informed do you feel as a member of your community about health care initiatives?
- What kind of views do you have about the facilities in your area?
- What influence do you think you have?
- What in your neighbourhood do you think contributes to good or bad health?
